# Thermal insulation does not hamper assessment of injuries in trauma CT scans

**DOI:** 10.1007/s10140-024-02272-8

**Published:** 2024-07-26

**Authors:** Tomasz Sanak, Aleksandra Skowronek, Konrad Mendrala, Tomasz Darocha, Grzegorz Liszka, Robert Chrzan, Krzysztof Jerzy Woźniak, Grzegorz Staskiewicz, Paweł Podsiadło

**Affiliations:** 1 Department of Emergency Medical Services, Jagiellonian University Medical College, Krakow, Poland; 2 Education and Medical Simulation Centre, Medical University of Silesia, Katowice, Poland; 3 Department of Anaesthesiology and Intensive Care, Medical University of Silesia, Katowice, Poland; 4 Laboratory of Radiological Measurements GL Center Ltd, Tychy, Poland; 5 Department of Radiology, Jagiellonian University Medical College, Kraków, Poland; 6 Chair and Department of Forensic Medicine, Jagiellonian University Medical College, Krakow, Poland; 7 Department of Clinical and Radiological Anatomy, Medical University of Lublin, Lublin, Poland; 8 Department of Emergency Medicine, Jan Kochanowski University, Kielce, Poland

**Keywords:** Accidental hypothermia, Emergency medicine, Pre-hospital care, Artifacts computed Tomography, Survival blankets, Insulation

## Abstract

**Purpose:**

The use of thermal insulations reduces the risk of hypothermia, therefore decreases the risk of death in trauma victims. The aim of the study was to assess whether thermal insulations cause artifacts, which may hinder the diagnosis of injuries, and how the used thermo-systems alter the radiation dose in polytrauma computed tomography.

**Methods:**

Computed tomography scans were made using the road accident victim body wrapped consecutively with 7 different covers. 14 injury areas were listed and evaluated by 22 radiologists. The radiation dose was measured using a dosimeter placed on the victim in the abdominal area.

**Results:**

No significant artifacts in any of the tested covers were observed. The presence of few minor artifacts did not hinder the assessment of injuries. Certain materials increased (up to 19,1%) and some decreased (up to -30,3%) the absorbed radiation dose.

**Conclusions:**

Thermal insulation systems tested in this study do not cause significant artifacts hindering assessment of injuries in CT scans. Concern for artifacts and increased radiation dose should not be a reason to remove patients’ thermal insulation during performing trauma CT-scanning.

## Introduction

Post-traumatic hypothermia is diagnosed with the decrease of core body temperature < 36 °C [1]. The main risk factors of post-traumatic hypothermia include traumatic brain injury, unclothing patients, the use of opioids and paralytic drugs, hemorrhagic shock, and Injury Severity Score > 15 [2–5]. Since hypothermia is an independent risk factor for mortality in trauma victims, a rigorous heat loss prevention should be provided [6, 7]. An algorithm for in-hospital hypothermia prevention and treatment in trauma victims has been proposed [2]. This includes careful thermal insulation and even active warming, correspondingly to patient’s core temperature. Scheck et al. in their study demonstrated that intrahospital transfers and performing an imaging examination in the computed tomography (CT) laboratory may significantly decrease core temperature in severely ill trauma patients. It should be stressed that the ambient temperature in a CT laboratory may be as low as 15 °C due to technical reasons [8]. Ong et al. in their study demonstrated that the drop in patient body temperature during CT imaging may peak up to 0.15 °C/min. [9]. However, some insulating covers and warming pads cause artifacts in CT scans and may increase radiation dose [10]. Since these findings were observed on phantoms, the impact of these artifacts on injuries visibility of a real patient remains unknown. To clarify the doubts, a similar study with a human body CT imaging was needed.

The aim of this study was to assess the occurrence of artifacts and alterations in radiation dose caused by insulating covers in CT imaging of trauma victims.

## Methods

### Study design

We conducted an experimental study. The body of road accident victim underwent trauma CT scanning postmortem in the Forensic Medicine Department. Siemens Somatom Emotion scanner was used. Victim’s body was placed on the Iron Duck spineboard (Iron Duck, Chicopee, MA, USA). The parameters of the CT protocol were as follows: tube voltage 130 kV; automated tube current; rotation time 0.6s; spiral pitch factor 0.8; slice thickness 1.5 mm.

### Image quality assessment

Initially, the scanning of victim’s body without insulation was done. Subsequently, victim was wrapped with the following layers of covers:

System 1: Metalized foil (MF) plus polyester blanket and MF (three-layers wrapping),

System 2: Blizzard Survival Blanket (Blizzard Protection Systems Ltd., Bethesda, UK),

System 3: Hypothermia Prevention and Management Kit (HPMK, North American Rescue, Greer, S.C., USA) and polyester blanket.

System 4: HPMK plus polyester blanket and Ready Heat heating pad (Tech Trade, Jersey City, NJ, USA),

System 5: Helios system (TacMed Solutions, Anderson, S.C., USA) and polyester blanket,

System 6: LESS Thermal Bag (Less AS, Kapp, Norway),

System 7: Mediwrap (Medical Innovations Group, Shoeburyness, Essex, UK).

An experienced radiologist assessed the images acquired without insulation to identify all injuries. Neighbouring injuries of the same type (e.g. fractures of adjacent ribs) were accumulated in 14 groups (injury areas) in order to facilitate further assessment, namely:

fractures of the spinous processes C5-Th1 (Area 1), fractures of the left transverse processes C6 and C7 (Area 2), luxation of the 1st left rib in costovertebral and costotransverse joints (Area 3), fractures of the left ribs namely 2nd in midaxillary line, 3rd in paravertebral region, 4th in posterior axillary line (Area 4), fractures of the left ribs 5th to10th between posterior axillary line and midaxillary line (Area 5), left clavicle fracture (Area 6), multiple fractures of the left scapula (Area 7), trachea disruption (Area 8), cervical muscles and vessels disruption in the left anterolateral region (Area 9), left pneumothorax (Area 10), fluid in the dorsal part of left pleura (Area 11), subcutaneous emphysema in the left epigastric and thoracic region (Area 12), blood-like shadows between the spleen and the left kidney (Area 13), irregular gas foci with hyperdense fluid zones in the 10th left lung segment (Area 14).

A uniform assessment sheet was prepared. Subsequently, all the images were assessed at radiological workstations independently by 22 radiologists. They were asked to describe appearance and intensity of artifacts in every injury area as well as in remaining regions. The artifact scale consisted of three grades: 0 – none, M – minor – artifacts are visible but do not hinder the image assessment, and S – significant – that may hinder the image assessment.

### Radiation dose assessment

A CT dose profiler PIRANHA Black 657 (RTI Electronics) was placed on the victim’s abdomen, similarly to the method described by Bauhs et al. [11]. A reference exposure value was measured while scanning without insulation. At every wrapping scenario, the dose was measured in the same way. Exposure values were compared to the reference in order to express the alteration of radiation dose caused by insulating covers.

### Statistical analysis

An analysis of the occurrence of artifacts for each system, both individually and collectively, was performed. The analysis was made separately for S-type artifact categories and M-type artifact categories. Each system and area were evaluated for artifacts against a reference group of scans (without the thermal insulation system). Pairwise analysis of nominal variables was based on 2 × 2 contingency tables using the McNemar’s test. In addition, the interobserver variability of all radiologists’ ratings was assessed using Cochran Q test. A *p* < 0.05 was accepted as statistically significant.

### Ethics

The consent of Jagiellonian University Ethical Board no 1072.6120.56.2018 was obtained.

## Results

### Image quality assessment

**Fig. 1:**
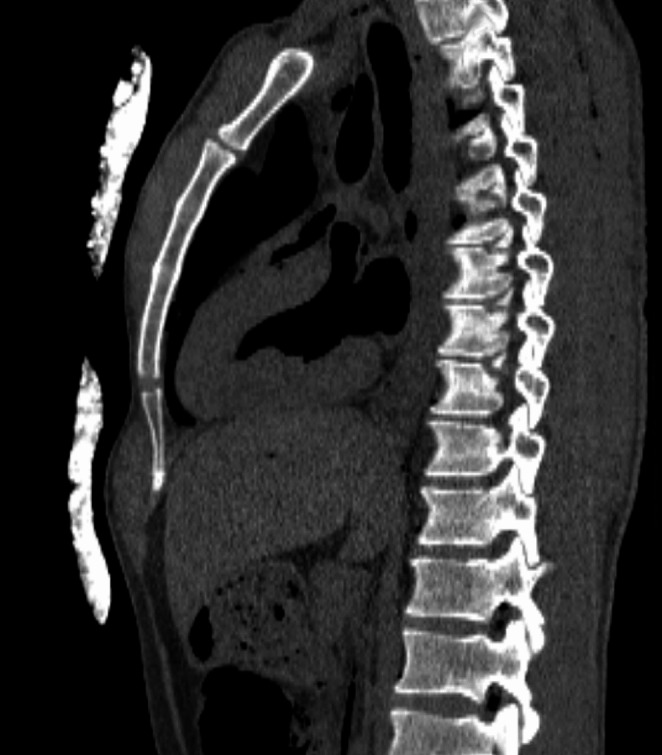
Heating panel arranged on the chest

**Fig. 2:**
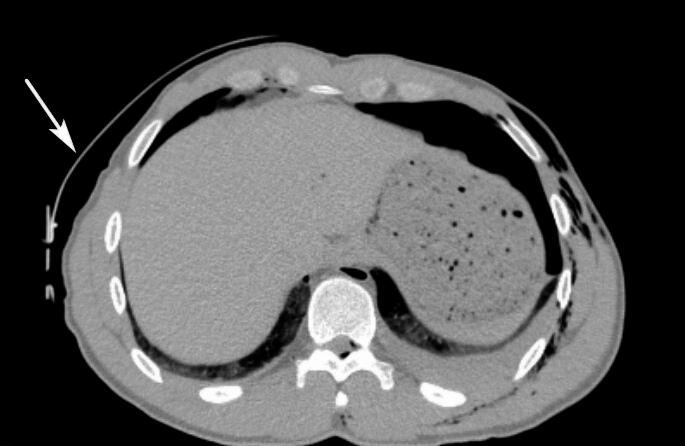
Less thermal bag insulation with fasternes (arrow)

No S-type artifacts hindering the CT images interpretation in any of the 7 thermo-systems and 14 areas were noticed. The assessments of all radiologists in this regard were identical. Warming pads and fastening elements of insulating systems did not veil patient’s tissues (Figs. 1 and 2).

**Fig. 3:**
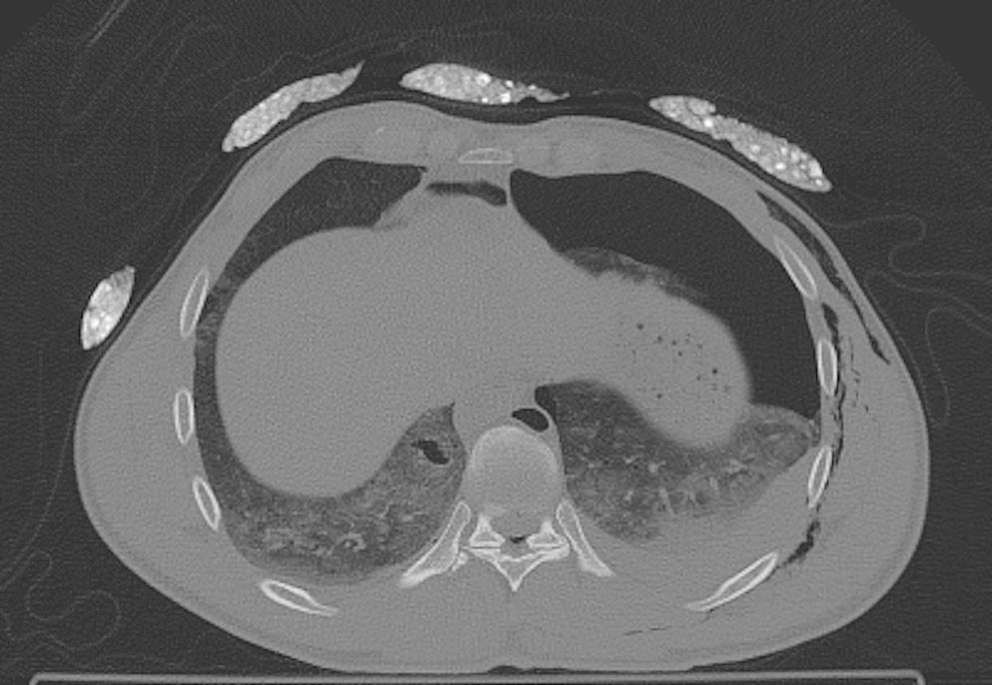
HPMK RH insulation system for 12 area

**Fig. 4:**
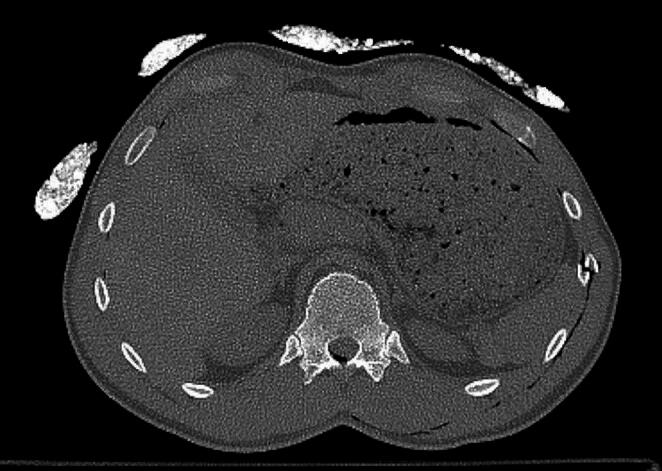
HPMK RH system for area 13 area

**Fig. 5:**
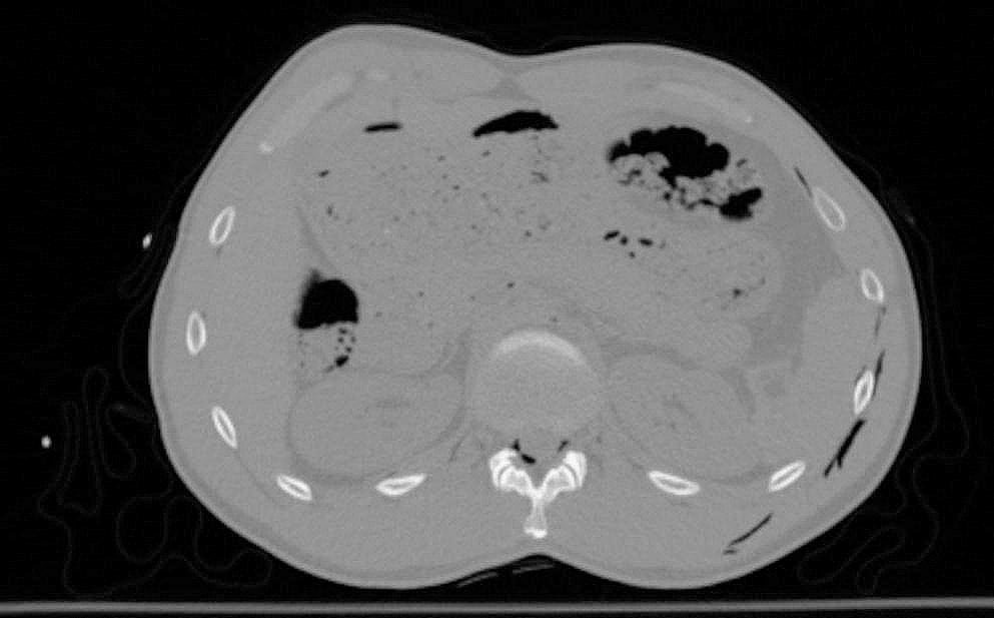
Helios insulation system for 13 area

The M-type artifacts were visible as subtle streak shadows, mainly in superficial tissues or outside of patient body. Occurrence of these artifacts was demonstrated with Systems 4, 5 and 6. Significant differences in ratings, compared to images without thermal insulation, were demonstrated in the area 12 for System 4 and in the area 13 for System 4 and System 5 (Figs. 3, 4 and 5). However, radiologists’ ratings for M-type artifacts were not consistent (*p* < 0.001).

Out of total 308 assessments for each thermal insulation, no artifacts were reported for System 1 and 7 (0%), six for System 2 (2%), four for System 3 (1%), 48 for System 4 (16%), 59 for System 5 (19%), and 58 for System 6 (19%).

### Radiation dose assessment

Insulating systems did alter the absorbed dose. The highest increase of radiation was caused by LESS, while the highest decrease was caused by HPMK with the Ready Heat warming pad. Details are summarized in Table 1.

**Table 1:** Differences in the radiation dose absorption for different thermal insulation systems

Insulation	Exposure – raw data (mGy)	CTDIvol (mGy)	Dose change (%)
No wrapping	4.78	7.29	---
**System 1** MF + polyester blanket + MF	4.46	6.72	-7.8
**System 2** Blizzard	4.57	6.98	-4.2
**System 3** HPMK + polyester blanket	5.6	8.58	17.7
**System 4** HPMK + polyester blanket + Ready Heat	3.29	5.08	-30.3
**System 5** Helios + polyester blanket	5.1	7.88	8.1
**System 6** LESS	5.42	8.68	19.1
**System 7** Mediwrap	4.89	7.54	3.4

## Discussion

Our study shows that using thermal insulation in trauma patients undergoing CT scanning does not cause artifacts hampering the assessment of injuries. Some minor artifacts may be caused by active warming pads and fastening elements of particular insulating systems. Although isolation systems change the absorbed dose, the increase in radiation is still acceptable.

A similar study was conducted by Podsiadło et al. [10]. Our results on image quality coincide with that part of aforementioned study that used automatic tube current modulation in abdominal protocol and image quality evaluation with a quality phantom. When the constant lamp current was used, artifacts were clearly visible. Thus, the automatic current adjustment in the standard test protocol eliminates artifacts caused by thermal insulations. Similar studies have been performed by Euler et al., Loewenhardt et al., Stokkeland et al., in which the absence of significant artifacts from the patient’s immobilization systems was proven [12–14]. Since some minor artifacts caused by insulating systems can occur, it seems to be beneficial that radiologists would be familiar with the structure of commonly used types of covers. This can reduce problems with image interpretation, such as pseudofractures described by Daffner et al. [15]

In this study, we found the alterations of absorbed radiation dose which slightly differ from those measured by Podsiadło et al. This can be caused due to two reasons. Firstly, we did not use a dosimetric cylinder. Instead, the CT dose profiler was placed on patient’s abdomen. This was removed and placed back on the patient each time when the insulating cover was changed. Therefore, its position to the patient’s tissues was similar, but not identical. Secondly, other CT parameters such as scan thickness and tube voltage were used. Despite some increases in exposure (max. 19.1%), the doses measured in this study are lower than those recommended for abdominal CT by the American College of Radiology (i.e.16 mGy), and the average dose used in Europe (25 mGy) [16, 17].

### Limitations of the study

Due to limited time frame for CT scanning, according to work schedule in Forensic Medicine Department, dose remeasurements was not possible. Hence, we could not calculate statistical significance of dose alterations.

Since we used the dose profiler which is intended for use with a cylinder phantom, doses calculated into CTDI_vol_ (Computed Tomography Dose Index) should be interpreted with caution.

## Conclusions

The thermal insulations tested in our study do not cause significant artifacts hampering assessment of injuries in polytrauma CT. Removal of warming pads or undoing the fastening system can be considered. None of the insulations tested increased the radiation dose to dangerous level.

## Data Availability

Data is available upon reasonable request addressed to the correspondent author and must be pre-approved by the bioethics committee of the Jagiellonian University Medical College.
